# Dual Challenge: Managing Complicated Diphtheria in a Child With Acute Myeloid Leukemia

**DOI:** 10.7759/cureus.94440

**Published:** 2025-10-13

**Authors:** Sudipto Bhattacharya, Hari Gaire, Aditi Tulsiyan, Anuj Singh, Abhishek Gupta, Saroj Dash, Sumi Nandwani, Anuj Sharma, Dharmendra K Singh, Manish Girhotra, Nita Radhakrishnan

**Affiliations:** 1 Department of Pediatric Hematology Oncology, Post Graduate Institute of Child Health, Noida, IND; 2 Department of Pediatric Otorhinolaryngology, Post Graduate Institute of Child Health, Noida, IND; 3 Department of Microbiology, Post Graduate Institute of Child Health, Noida, IND; 4 Department of Pediatric Cardiology, Post Graduate Institute of Child Health, Noida, IND; 5 Department of Pediatrics, Post Graduate Institute of Child Health, Noida, IND

**Keywords:** acute myeloid leukemia, cardiac dysfunction, diphtheria, immunocompromised host, pediatric oncology, vaccine-preventable diseases

## Abstract

Acute myeloid leukemia (AML) is an aggressive childhood malignancy that is associated with increased risk of infections and toxic deaths, particularly in low- and middle-income countries (LMICs). In febrile neutropenia, the predominant pathogens are Gram-negative bacteria, although Gram-positive organisms such as *Staphylococcus aureus* are also commonly encountered. Diphtheria, caused by *Corynebacterium diphtheriae*, although controlled by universal immunization programs of the government, is reported sporadically. We report the occurrence of such a fatal infection at the initial presentation of AML. A 10-year-old boy presented with fever, sore throat, gum swelling, poor oral hygiene, and pallor for a three-day duration. He was subsequently diagnosed with acute myeloid leukemia and was started on induction therapy. The baseline echocardiography showed cardiac dysfunction, and hence, he was managed with modified induction with cytarabine and etoposide only and no anthracyclines. As part of the febrile neutropenia protocol, he was started on antibiotics and antifungals. The complaint of sore throat progressed to membranous tonsillitis within a week. This was swabbed and was found to be positive for *Corynebacterium diphtheriae*. His further course was complicated by septic shock, myocarditis, and prolonged neutropenia. He was managed with induction chemotherapy for AML, broad-spectrum antimicrobials, anti-diphtheritic serum, granulocyte transfusions, antifungals, and cardiac support. Although he achieved remission from AML and recovered from diphtheria-related complications, the left ventricular dysfunction persisted. He remained positive for measurable residual disease for which he received three lines of therapy in view of refractory AML; in view of non-responsive AML, he was palliated and the family opted to discontinue therapy. To the best of our knowledge, this is the first reported case of pediatric AML, associated with complicated *Corynebacterium diphtheriae* infection. A review of literature reports the rarity and diagnostic and treatment options in this scenario. It also underscores the need for vigilance regarding vaccine-preventable diseases in pediatric oncology. We also discuss the need for prophylaxis of close contacts. The complicated and abridged induction may have contributed to the persistence of disease and eventual poor outcome in this child. The case underscores the dual challenge of managing AML and treating diphtheria infection, further complicated by myocarditis with left ventricular dysfunction and prolonged neutropenia. Prompt anti-diphtheritic serum helped in defervescence and reversal of symptoms.

## Introduction

Acute myeloid leukemia (AML) is an aggressive malignancy which accounts for approximately 15%-50% of all pediatric leukemias. In high-income countries, the survival rates approach 70%, whereas the outcome in low- and middle-income countries (LMICs) remains suboptimal. A major reason for this is treatment-related toxicity due to infections, hemorrhage, or tumor lysis [1]. Although most infections encountered during cancer treatment are nosocomial or opportunistic in nature, in LMICs, especially in young children, vaccine-preventable infections also contribute to the morbidity. Children who receive cytotoxic chemotherapy or other immunosuppressive therapies frequently encounter measles, varicella, herpes zoster, influenza, among others due to their impaired humoral and cellular immune responses. These are noted to be disseminated, resulting in organ toxicity such as pneumonia, hepatitis, encephalitis, etc. [2,3]. This not only adds to morbidity but also increases treatment breaks for cancer, thus increasing risk of poor disease outcome. The risk of vaccine-preventable infections could be due to incomplete primary immunization, poor coverage of primary immunization, or even due to waning vaccine-induced immunity during or after chemotherapy [4]. In LMIC setting, community or household outbreaks of such infections warrant secondary prophylaxis for patients to reduce their risk for infection and dissemination. Also, re-immunization should be completed post-cancer treatment before integration of the child back into the society [2].

Diphtheria is a vaccine-preventable infection caused by *Corynebacterium diphtheriae* that produces a fibrinous pseudomembrane in the upper airway and systemic manifestations such as peripheral neuritis and myocarditis. Although inclusion of diphtheria in child immunization has rendered classical respiratory diphtheria uncommon now, the disease still exists as a public health threat in many parts of the world, especially in vulnerable groups such as children, elderly, and cancer patients [5]. Immunosuppressed patients are at heightened risk for both typical infections as well as atypical complications due to the partial protection offered by prior immunization and the loss of vaccine-derived antibody titers during immunosuppression. The loss of protective antibody to diphtheria has been reported commonly, thus warranting appropriately timed re-immunization in those who complete treatment [6].

In the last decade, India has been a focal point for attention on diphtheria, due to its share of notable outbreaks. Several thousands of cases were reported in 2020-2021, with epidemiological shifts to older children and adolescents, as well as elderly due to waning immunity after primary infection and frequent missing of booster dose during school age [7]. Despite this, published reports of diphtheria in pediatric cancers is limited [5,8-10]. In this report, we discuss the difficulties associated with co-diagnosing acute diphtheritic pharyngitis with pseudomembrane and AML in an older child, the complications of the infection, and outcome of both the infection and the malignancy.

## Case presentation

A 10-year-old boy presented to us with complaints of fever, sore throat for seven to 10 days, fatigue, gum swelling, and pallor. His sore throat was insidious, with difficulty in swallowing food. This was associated with the paleness of the body reported by parents, for which they sought medical consultation. He has had no prior hospitalizations; he was vaccinated for age till five years; however, his 10-year booster for tetanus-diphtheria-acellular pertussis (Tdap) vaccine was missed. He was evaluated at the primary center, where blood counts were shown to be low, and hence, he was referred to us for further evaluation for leukemia. At admission, he was febrile and tachycardic, with a blood pressure of 110/70 mm Hg. He had gingival hyperplasia, congested pharynx, and levels 1 and 2 cervical lymphadenopathy and hepatosplenomegaly at admission. Cardiac and neurological clinical examination was normal at admission. He was maintaining saturation in room air, with no airway obstruction. Anthropometry showed low weight for age. There was no evidence of cardiac dysfunction or neurological abnormality at admission. Complete blood count revealed pancytopenia with circulating blasts. Flow cytometry done from peripheral blood revealed blasts positive for CD13, CD33, CD64, CD117, CD34, CD38, and CD45, while myeloperoxidase was negative. Based on the diagnosis, he was started on AML induction, with Cytarabine (AraC) + Daunorubicin + Etoposide (ADE) regimen: cytarabine (3 days), daunorubicin (5 days), and etoposide (10 days) under antibiotics cover. Baseline cardiac evaluation was normal.

**Figure 1:**
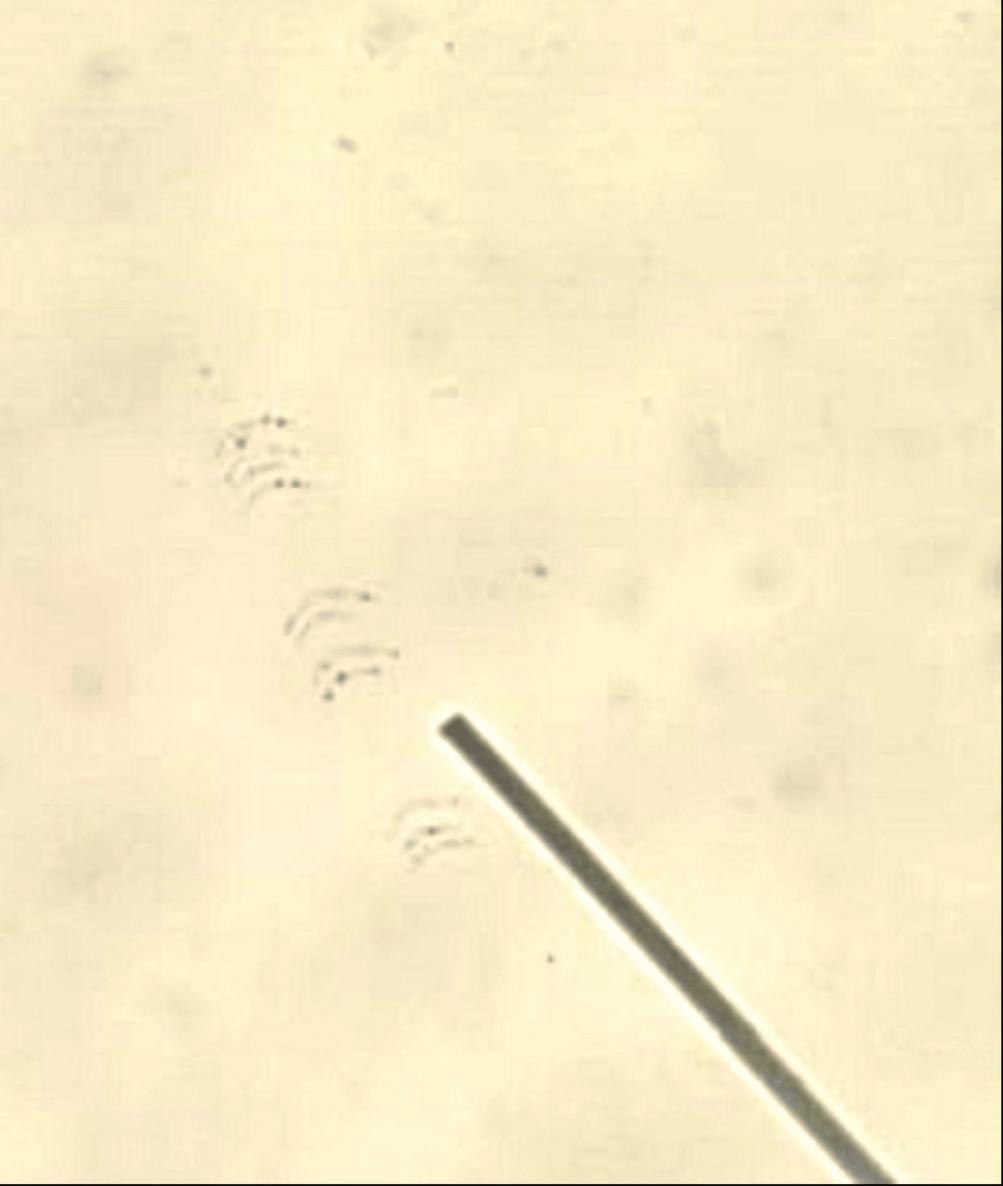
Gram-positive rods with metachromatic granules suggestive of infection with Corynebacterium diphtheriae

During induction, he continued to be febrile. By day 8, there was persistence of fever with hypotension requiring fluid boluses, escalation of antibiotics and antifungals and inotropes. Standard infection screen for febrile neutropenia including blood culture and sensitivity (CS) and chest CT was negative. Dengue serology (IgM) was positive. He developed membranous tonsillitis, which on attempted removal during otolaryngology consult showed bleeding spots. The membrane was partially removed and on microbiological examination noted to have Gram-positive rods with metachromatic granules, consistent with *Corynebacterium diphtheriae* (Figure 1).

There was no history of contact with an active case of diphtheria, either prior to hospital presentation or during admission. The diagnosis was confirmed by culture. Anti-diphtheritic serum (ADS) was administered promptly, along with supportive measures at a dose of 90,000 IU as a single infusion after sensitivity testing [11]. The details of all investigations have been compiled in Table 1.

**Table 1:** Laboratory evaluation AML: acute myeloid leukemia, MRD: measurable residual disease, CECT: contrast-enhanced computed tomography, EF: ejection fraction.

Parameter	Report	Normal Reference Range (pediatric)
Hemoglobin (Hb)	10.7 g/dl	11.5-15.5 g/dL
White blood cell count (WBC)	329×10⁹/L	4.5-13 ×10⁹/L
Platelet count	68×10⁹/L	150-450 ×10⁹/L
Peripheral smear	Hyperleukocytosis with myeloblasts and thrombocytopenia	-
Flow cytometry	Blasts positive for CD13, CD33, CD64, CD117, CD34, CD38, CD45	-
Molecular panel for AML	Negative for common transcripts	
Bone marrow MRD	Positive (0.45%)	Negative (<0.1%)
Dengue serology	IgM positive	Negative
Blood culture	Negative	Negative
Throat swab culture	No growth	
CECT chest (for fungal infection)	Negative	Negative
Throat swab (Albert’s stain)	Gram-positive rods	Negative
Echocardiogram (Baseline)	Normal	Normal EF >55%
Echocardiogram (Day 14)	Systolic dysfunction, EF 45%	Normal EF >55%
Electrocardiogram	Normal sinus rhythm	Normal sinus rhythm

**Figure 2:**
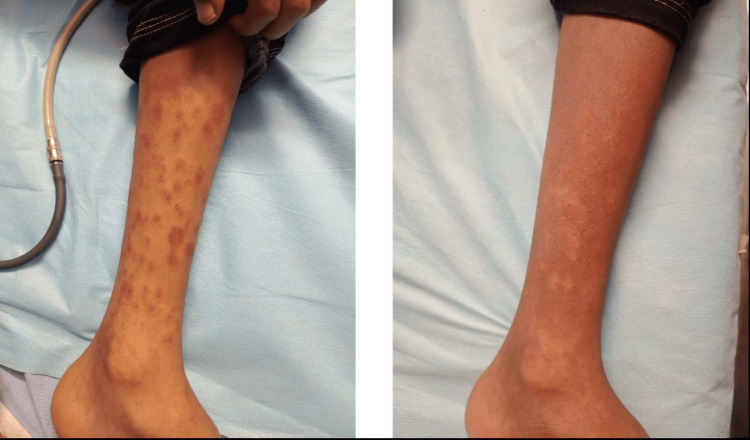
Diffuse maculopapular rash with erythematous base

During this week, he also developed a diffuse maculopapular rash appeared on the trunk, likely diphtheria related (Figure 2).

**Figure 3:**
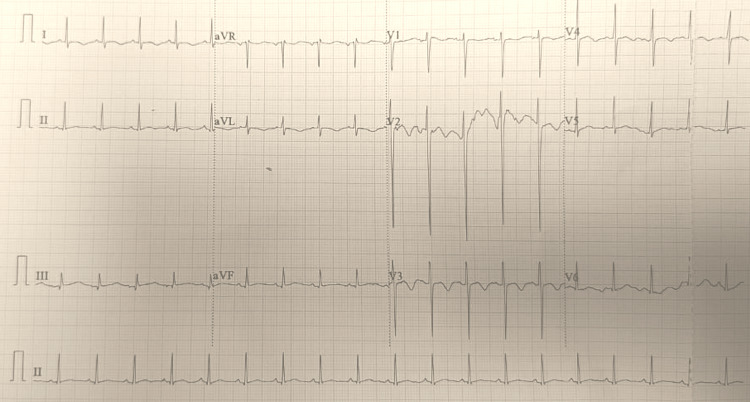
A 12-lead electrocardiogram with normal standardization, showing heart rate of 120 beats per minutes, normal p wave, and QRS axis aVR: augmented voltage right arm, aVL: augmented voltage left arm, aVF: augmented voltage foot (left leg).

In view of severe thrombocytopenia, skin biopsy was not attempted. He improved with the administration of ADS with defervescence and improvement in blood pressure records. Repeat echocardiogram was done to rule out cardiac involvement due to diphtheria; this evaluation done after 14 days from the baseline showed systolic dysfunction with an ejection fraction of 45%. He also developed nasal regurgitation requiring nasogastric feeding by the third week of admission. He was managed with supportive care for both diphtheritic myocarditis with carvedilol, enalapril and carnitine, with aggressive nutritional support and airway protection. Dose titration of carvedilol was done with cardiac evaluation with ECG daily and echocardiogram once weekly. ECG showed normal sinus rhythm with normal p wave and QRS axis, without any evidence of heart blocks (Figure 3).

He remained neutropenic till fourth week of induction, which was managed with antimicrobials, platelet, and granulocyte support. The skin rash coalesced after ADS administration and resolved after three to four weeks. Close contacts of this patient including parents, nurses, and doctors were advised chemoprophylaxis with erythromycin or azithromycin [12]. There was no spread to any patient or healthcare professional in the ward. Partially vaccinated healthcare professionals were advised to complete booster dose of diphtheria. The child was managed in a separate room with no contact to other patients during the entire period.

Once counts recovered, he underwent disease response assessment; bone marrow was in morphological remission; however, measurable residual disease (MRD) was positive (0.45%, negative <0.1%). Over the next two months, he had a complete neurological recovery; cardiac function improved steadily, and he was weaned off cardiac medications. In view of positive MRD, the family was counseled for allogeneic hematopoietic stem cell transplantation after salvage chemotherapy. As conventional salvage protocols in AML contained anthracyclines such as idarubicin were not feasible in this child due to cardiac dysfunction, he was treated with one cycle each of high-dose cytarabine (18 g/m^2^), venetoclax-azacitidine, and fludarabine, cytarabine, granulocyte colony-stimulating factor (GCSF) (FLAG)-bortezomib. He continued to be refractory to treatment and was palliated subsequently.

## Discussion

National and international surveillance data on diphtheria have reported episodic surges in India. These breakthrough infections in previously vaccinated persons have demonstrated an epidemiological change of age shift, with older children and young adults being affected rather than children under five. These outbreaks have been reported mainly in localized communities with vaccine hesitancy, poor primary vaccination, and poor booster uptake, resulting in high case fatality rates [13]. These highlight the fact that diphtheria remains a public health concern in India and other LMICs. From a policy and a clinical standpoint, the re-emergence of preventable infections suggests strengthening adolescent booster programs and revaccination of those who may have missed completing the vaccination schedule [14].

Both toxigenic and non-toxigenic invasive variants have been reported to cause severe local and systemic infection such as cutaneous disease and endocarditis [14]. Severe cutaneous infection has been reported in immunocompromised and post-surgical patients. Our patient had history of missing the second booster dose of diphtheria which is recommended at 10 years of age as per universal immunization program and developed a breakthrough infection with both local and systemic features such as membranous tonsillitis, disseminated skin rash, myocarditis, and nasal regurgitation.

Patients with cancer, especially children receiving intensive chemotherapy for hematological malignancies, are at risk for both typical and atypical infectious complications. As in breakthrough infections in immunocompetent, waning immunity due to fall in antibody titers developed during primary immunization is important in the context of cancer as well. In addition, the reduction in cell-mediated and humoral immunity during cancer treatment may also play a role, especially in patients who have been on long-term immunosuppression [6]. Although outbreaks of diphtheria have been reported, there is hardly any published literature on managing diphtheria and malignancy together; the challenges expected. The few cases that have been reported of toxigenic and non-toxigenic diphtheria, their presentations, and outcome have been compiled as Table 2 [5,9,11,15,16].

**Table 2:** Summary of published cases of Corynebacterium diphtheriae in patients with malignancies ADS: anti-diphtheritic serum.

Serial Number	Year	Author	Age	Underlying Malignancy	Clinical Presentation	Organism Toxigenicity	Outcome
1	2001	Mattos-Guaraldi et al., [5]	45 years	Infiltrating basaloid carcinoma	Ulcerated lesion	Toxigenic *C. diphtheriae*	Not reported
2	2009	Gomes et al., [15]	Adult (not specified)	Bladder cancer with nephrostomy catheter	Catheter-related infection	Toxigenic *C. diphtheriae*	Not reported
3	2012	Wojewoda et al., [12]	23 years	Acute myeloid leukemia	Bloodstream infection	Non-toxigenic *C. diphtheriae*	Recovered from infection. Treated with antibiotics.
4	2015	Lake et al., [9]	3 years	Acute lymphoblastic leukemia	Necrotizing epiglottitis requiring urgent intervention	Non-toxigenic *C. diphtheriae*	Recovered from infection. Treated with antibiotics and ADS.
5	2022	Abdolkarimi et al., [16]	15 years	Osteosarcoma (post-surgical)	Post-surgical site infection with cutaneous diphtheria	Non-toxigenic *C. diphtheriae*	Recovered from infection. Treated with antibiotics and ADS.
6	Present case 2025	Bhattacharya et al., 2025	10 years	Acute myeloid leukemia	Tonsillitis, myocarditis, neuritis, skin rash	Toxigenic *C. diphtheriae*	Recovered from infection. Treated with antibiotics and ADS.

It is noteworthy that both toxigenic and non-toxigenic strains have been reported in patients with cancer, presenting with a wide range of clinical manifestations from localized/surgical site infections to blood stream infections. Cardiac involvement is the primary cause of acute mortality in classical diphtheria [17,18]. Multiple reports have highlighted the occurrence of myocarditis and conduction abnormalities in the days to weeks after onset of pharyngeal disease. In our case, the myocarditis was associated with abnormal cardiac function, febrile neutropenia, and sepsis and it precluded us from using anthracyclines in the further treatment of this child. However, myocarditis was stable with aggressive supportive care, and over a period of next two months, cardiac function improved.

Our case highlights few salient features of coexistent diphtheria and AML. Prompt diagnosis is crucial. In children with prolonged neutropenia as encountered in many cancers including AML, the presence of mucositis with throat infection is not unusual. In most cases, we tend to treat with broad-spectrum antibiotics and antifungals, as per standard febrile neutropenia guidelines [19]. Since diphtheria and other vaccine-preventable infections are not common in high-income countries, the index of suspicion is often low. While treating patients in LMICs, especially in communities with vaccine hesitancy, it would be prudent to take a full immunization history at the outset and evaluate for such infections in case of non-responsive fevers. Secondly, early administration of anti-diphtheritic serum was the cornerstone to recovery of this child from diphtheria, despite the disseminated course with neurological and cardiac manifestations. Finally, supportive care for airway, cardiac management, transfusion support, and nutritional rehabilitation helped in managing the child with significant comorbidities. The fact that further chemotherapy was compromised due to inability to use anthracyclines might have contributed to the poor outcome in AML. This again highlights the need to complete vaccinations including boosters, so as not to be infected with such organisms that are completely preventable.

## Conclusions

To summarize, the evidence on diphtheria occurrence during cancer is largely anecdotal. There are no large case series describing clinical heterogeneity, management, and outcome in this cohort. Existing microbiological evidence points that both toxigenic and non-toxigenic strains can cause severe and invasive infections in patients with malignancies. Clinicians treating cancer, in areas where diphtheria is prevalent or in case of recent travel from an outbreak region, should maintain a high index of suspicion for membranous tonsillitis, unexplained myocarditis, neurological findings, and airway disease. The prompt administration of anti-serum along with antibiotics and supportive care remains the mainstay of treatment. Literature review confirms the extreme rarity of such cases, but the implications for clinical practice and public health are significant.
